# [Retracted] Upregulation of cervical carcinoma expressed PCNA regulatory long non-coding RNA promotes esophageal squamous cell carcinoma progression 

**DOI:** 10.3892/ol.2025.15380

**Published:** 2025-11-10

**Authors:** Xiaojun Wang, Liangfen Zhou, Huiyun Zhang, Hui Ou, Wenxing Long, Xiaobao Liu

Oncol Lett 20: 142, 2020; DOI: 10.3892/ol.2020.12006

Following the publication of this paper, it was drawn to the Editor's attention by a concerned reader that the flow cytometric assay data shown in Fig. 3A on p. 5 had already appeared in a pair of previously published articles written by different authors at different research institutes. A subsequent analysis performed by the Editorial Office revealed that western blot data included in Figs. 3 and 5 and the migration and invasion assay data in Fig. 3C and D had also appeared in previously published articles written by different authors at different research institutes.

Owing to the fact that the contentious data in the above article had already been published prior to its submission to *Oncology Letters*, the Editor has decided that this paper should be retracted from the Journal. The authors were asked for an explanation to account for these concerns, but the Editorial Office did not receive a reply. The Editor apologizes to the readership for any inconvenience caused.

